# Association between nut consumption and metabolic syndrome in Korean adults: results from the Korean Genome and Epidemiology Study–Health Examinees

**DOI:** 10.3389/fnut.2024.1400212

**Published:** 2024-05-23

**Authors:** Hye Ran Shin, SuJin Song

**Affiliations:** Department of Food and Nutrition, Hannam University, Daejeon, Republic of Korea

**Keywords:** nut, metabolic syndrome, KoGES, Korean adults, cardiovascular disease

## Abstract

**Background:**

The epidemiological evidence regarding nut consumption and metabolic diseases focuses on Western populations. Nut consumption among Koreans is relatively low, and the prevalence of metabolic syndrome is rapidly increasing, highlighting the need for more focused studies in this population. This cross-sectional study aimed to investigate the relationship between nut consumption and metabolic syndrome in Korean adults.

**Methods:**

A total of 112,501 adults (39,481 men and 73,020 women) aged 40–79 years were selected from baseline data of the Korean Genome and Epidemiology Study–Health Examinees. Nut consumption was assessed using a validated semi-quantitative food frequency questionnaire and categorized as non-consumers, <1 serving/week, ≥1 to <2 servings/week, or ≥2 servings/week (15 g per serving). Metabolic syndrome and its components were defined according to the Korean Society of CardioMetabolic Syndrome criteria. Associations between nut consumption and metabolic syndrome and its components were examined using multiple logistic regression with adjustments for potential confounders.

**Results:**

In the study population, the prevalence of metabolic syndrome was 21.4% (26.9% in men and 18.4% in women), and the mean nut intake was 0.8 serving/week (0.7 serving/week in men and 0.8 serving/week in women). After adjusting for confounders, higher nut consumption was associated with a lower odds ratio (OR) of metabolic syndrome in individuals consuming ≥2 servings/week compared with non-consumers [OR = 0.85, 95% confidence interval (CI) = 0.80–0.91, *p* for trend <0.001]. Specifically, in men, this level of consumption was associated with a 14% reduction in the OR of metabolic syndrome (OR = 0.86, 95% CI = 0.77–0.95, *p* for trend = 0.028). In women, a similar reduction of 14% was observed (OR = 0.86, 95% CI = 0.80–0.93, *p* for trend <0.001). Among the metabolic syndrome components, nut consumption was inversely associated with abdominal obesity, low high-density lipoprotein-cholesterol, and elevated triglycerides in men and women, whereas no associations were observed for elevated blood pressure or elevated fasting blood glucose.

**Conclusion:**

Our findings suggest that higher nut consumption is inversely associated with metabolic syndrome and its components in Korean adults. Further studies are needed to examine the longitudinal association between nut consumption and metabolic diseases in this population.

## 1 Introduction

Metabolic syndrome is defined as a combination of cardiometabolic risk factors that increase the risk of serious health problems such as cardiovascular disease (CVD) and type 2 diabetes (1, 2). The National Cholesterol Education Program (NCEP) Adult Treatment Panel III (ATP III) diagnoses metabolic syndrome in individuals with three or more of the following components: elevated waist circumference, blood pressure, fasting blood glucose and triglycerides, and reduced high-density lipoprotein (HDL)-cholesterol (3). The development of metabolic syndrome is closely related to an unhealthy lifestyle (4).

Metabolic syndrome and its components are closely associated with dietary patterns and nutrient and food intake (5–7). To prevent metabolic diseases such as hypertension, type 2 diabetes, metabolic syndrome, and CVD, two dietary approaches, the Dietary Approaches to Stop Hypertension (DASH) and the Mediterranean dietary pattern, have been recommended (8, 9). Both of these healthy dietary patterns include nuts as one of the major food components because nuts are a good source of monounsaturated and polyunsaturated fatty acids, dietary fiber, vitamins E and K, and minerals such as magnesium, copper, potassium, selenium, carotenoids, and antioxidants (10).

Research data on nut consumption and metabolic syndrome are accumulating; however, findings on the association between nut consumption and metabolic syndrome and its components are inconsistent. A meta-analysis of prospective cohort studies revealed that an increase of 1 serving (30 g) per week of nuts decreased the risk of metabolic syndrome by 4% (11) but did not report an association between nut consumption and each metabolic syndrome component. Two additional meta-analyses of randomized controlled trials demonstrated that nut intake had favorable effects on some metabolic syndrome components, such as serum triglycerides and fasting blood glucose, whereas no effect on other metabolic syndrome components was observed (12, 13).

Furthermore, most research is concentrated in Western countries, and few studies have been conducted in Asia. Two studies on Korean adults reported inconsistent findings. A prospective cohort study reported that consuming >15 g of nuts per week was inversely associated with the incidence of metabolic syndrome in Korean adults (14), but did not examine the association of nut consumption with metabolic syndrome components. A randomized controlled trial among Korean patients with metabolic syndrome revealed that nut consumption did not improve the levels of markers of metabolic syndrome (15). Therefore, it is necessary to investigate the association of nut consumption with metabolic syndrome and its components in a larger Korean cohort sample to better understand the effect of nut consumption on metabolic syndrome in this population.

The overall age-adjusted prevalence of metabolic syndrome in Korea has increased from 27.1% in 2001 to 33.2% in 2020 (16). According to data on the causes of death from Statistics Korea, heart and cerebrovascular diseases were the second and fourth leading causes of death, respectively (17). Therefore, it is necessary to identify the dietary factors related to metabolic syndrome and incorporate them into dietary guidelines to improve cardiometabolic health in Korean adults. To fully understand the association of nut consumption with metabolic syndrome and its components, this study analyzed the association between nut consumption frequency and metabolic syndrome using a large dataset of Korean adults.

## 2 Materials and methods

### 2.1 Data and study participants

This study used baseline data from the Korean Genome and Epidemiology Study-Health Examinees (KoGES-HEXA). The KoGES-HEXA is a population-based, large-scale cohort study aimed at identifying environmental and genetic risk factors for major chronic diseases prevalent among Koreans (18). The KoGES-HEXA commenced its baseline survey in 2004 and has been conducting follow-up surveys. It focuses on city regions, centering around medical institutions, health centers, and public health clinics. The study participants were Korean men and women over 40 years who had visited medical institution screening centers in urban areas. The KoGES-HEXA collected data on sociodemographic and medical history, lifestyle, anthropometric measures and biochemical variables, and dietary variables (19).

The baseline data of the KoGES-HEXA recruited 173,195 men and women aged 40–79 years from urban medical institutions across South Korea between 2004 and 2013. From this group, 3,064 individuals who did not complete the food frequency questionnaire (FFQ) were excluded. Additionally, 834 participants whose daily energy intake calculated from the FFQ was <500 kcal or >5,000 kcal were excluded (20–22). Subsequently, 35,769 individuals who did not provide data on variables relevant to the study were excluded (2,562 for physical measurements; 26,105 for education, occupation, and household income; 555 for smoking, alcohol consumption, and physical activity; and 6,547 for biochemical measurements). Finally, 147 individuals who did not respond to questions about the diagnoses of hypertension, diabetes, and hyperlipidemia and 20,880 individuals who did not respond to questions about medication use for these conditions (hypertension, diabetes, or dyslipidemia) were also excluded. Finally, the data of 112,501 participants (39,481 men and 73,020 women) were analyzed. All study participants provided written informed consent. This study qualified for an exemption issued by the Institutional Review Board of Hannam University (2023-E-01-09-0625).

### 2.2 Assessment of nut consumption and nutrient intake

Dietary data collected using the validated semi-quantitative FFQ were used to assess nut consumption (23). The FFQ asked participants how often they had consumed nuts, including peanuts, almonds, and pine nuts, in the past year. The responses to this question comprised nine consumption frequency options (rarely, 1 time/month, 2–3 times/month, 1–2 times/week, 3–4 times/week, 5–6 times/week, 1 time/day, 2 times/day, and 3 times/day) and three average serving sizes per consumption (0.5, 1, and 1.5 servings). This study converted the responses into a weekly consumption frequency of nuts based on one serving (15 g) for each participant and categorized nut consumption frequency into non-consumers, <1 serving/week, 1–2 servings/week, and ≥2 servings/week, considering the distribution of participants in each category of consumption frequency.

Energy and nutrient intakes were evaluated using dietary data obtained from the FFQ. The percentage of energy from each nutrient was calculated for carbohydrate, protein, and fat intake. Vitamin and mineral intakes were evaluated as nutrient intake per 1,000 kcal/day to assess the quality of nutrient intake.

### 2.3 Definition of metabolic syndrome and its components

The anthropometric measurements used in this study included height, weight, body mass index (BMI), waist circumference, systolic blood pressure (SBP), and diastolic blood pressure (DBP). Height and weight were measured using an automatic height-weight measuring device in centimeters (cm) and kilograms (kg), respectively. BMI was calculated as measured weight divided by the square of measured height in meters (kg/m^2^). Waist circumference was measured in centimeters using a measuring tape. The SBP and DBP were measured twice at the arm at a minimum interval of 1 min, and the average of the two measurements was used. Biochemical tests were conducted according to the standardized protocols of the participating medical institutions; the biochemical indicators were total cholesterol, triglycerides, HDL-cholesterol, low-density lipoprotein (LDL)-cholesterol, and fasting blood glucose. Blood tests were performed on venous blood samples collected after fasting for >8 h. Total cholesterol, triglyceride, HDL-cholesterol, and fasting blood glucose levels were measured using enzymatic methods with the ADVIA 1650 System (Siemens, Tarrytown, NY, United States). The LDL-cholesterol level was calculated using the Friedewald formula (total cholesterol − HDL-cholesterol – [triglycerides ÷ 5]).

Metabolic syndrome was defined according to the criteria set by the Korean Society of Cardio-Metabolic Syndrome. Metabolic syndrome was identified as the presence of three or more of the following conditions: abdominal obesity (waist circumference ≥ 90 cm in men and ≥85 cm in women), low HDL-cholesterol (<40 mg/dL in men and <50 mg/dL in women or medication use), elevated triglycerides (≥150 mg/dL), elevated blood pressure (SBP ≥ 130 mmHg or DBP ≥ 85 mmHg or medication use), and elevated fasting blood glucose (≥100 mg/dL or medication use) (24, 25).

### 2.4 Measurement of other variables

General characteristics of the participants included age, educational level, and household income. Educational level was categorized as middle school or lower, high school or lower, and college or higher. Household income was classified as <2 million Korean Won (KRW) per month, <4 million KRW per month, or ≥4 million KRW per month. The lifestyle variables included smoking status, alcohol consumption, and physical activity. Smoking status was categorized as current smoker, former smoker, or non-smoker. Alcohol consumption was categorized as current drinkers, former drinkers, and non-drinkers. Physical activity was assessed based on the response to the question, “Do you regularly perform exercise intensely enough to cause sweating?” and classified as “yes” or “no” (26, 27).

### 2.5 Statistical analyses

Statistical analyses were performed using SPSS software (version 25.0; IBM Corp., Armonk, NY, United States). General characteristics were presented as mean and standard error for continuous variables and as frequencies and percentages for categorical variables. Differences in general characteristics according to nut consumption frequency were evaluated using analysis of variance for continuous variables and the chi-square test for categorical variables. Differences in energy and nutrient intake, anthropometric measurements, and biochemical test results according to nut consumption frequency were assessed using multiple regression analysis after adjusting for covariates (sex, age, educational level, household income, smoking status, alcohol consumption, physical activity, BMI, and energy intake, where applicable). The associations between nut consumption frequency and metabolic syndrome and its components were analyzed using multiple logistic regression to calculate the odds ratios (ORs), 95% confidence intervals (CIs), and *p*-values for trend. In this analysis, the reference group was comprised of non-consumers. The potential confounding variables adjusted for were sex, age, educational level, household income, smoking status, alcohol consumption, physical activity, BMI, and energy intake. Statistical significance was set at *p* < 0.05.

## 3 Results

### 3.1 Distribution of nut consumption frequency

**Figure 1:**
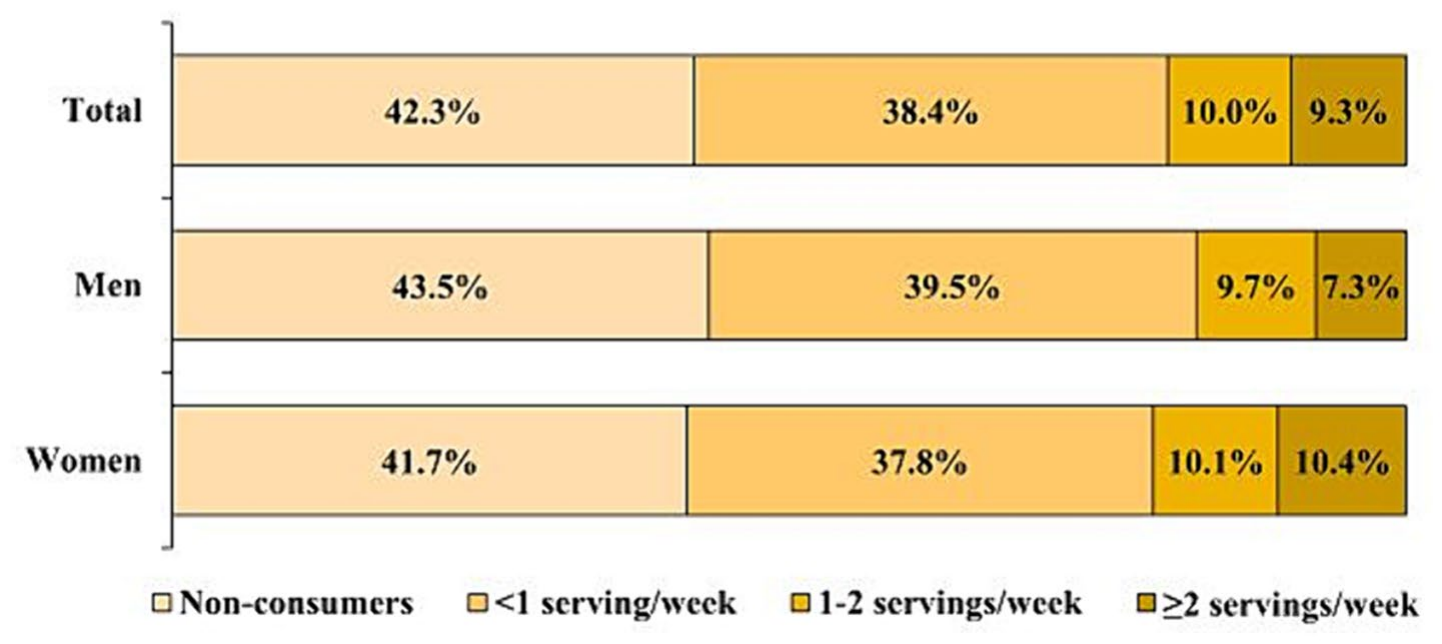
Percentage of subjects in each nut consumption frequency category.

The distribution of nut consumption frequency is illustrated in Figure 1. Among all participants, 42.3% were non-consumers, 38.4% consumed <1 serving/week, 10.0% consumed 1–2 servings/week, and 9.3% consumed ≥2 servings/week. Among men, 43.5% were non-consumers, 39.5% consumed <1 serving/week, 9.7% consumed 1–2 servings/week, and 7.3% consumed ≥2 servings/week. Among women, 41.7% were non-consumers, 37.8% consumed <1 serving/week, 10.1% consumed 1–2 servings/week, and 10.4% consumed ≥2 servings/week. The mean nut intake was 0.8 serving/week among all participants (0.7 serving/week in men and 0.8 serving/week in women).

### 3.2 Characteristics of the study participants according to nut consumption frequency

The general characteristics of the study participants according to their nut consumption frequency are presented in Table 1. The average age of participants was 53.0 years (53.8 years in men and 52.6 years in women). In both men and women, the highest nut consumption group had a higher proportion of individuals with higher levels of education and household income, non-smokers, and non-current alcohol users, and individuals who engaged in physical activity (*p*-values <0.001). The prevalence of metabolic syndrome in the study population was 21.4% (26.9% in men and 18.4% in women). Compared with the group of non-consumers, the prevalences of metabolic syndrome and its components were lower in the group consuming ≥2 servings/week of nuts (*p*-values <0.001).

**Table 1:** Characteristics of the study subjects according to nut consumption frequency.

		Total					Men					Women				
		Non-consumers	<1 serving/week	1–2 servings/week	≥2 servings/week	*p*-value	Non-consumers	<1 serving/week	1–2 servings/week	≥2 servings/week	*p*-value	Non-consumers	<1 serving/week	1–2 servings/week	≥2 servings/week	*p*-value
n		47,602	43,164	11,227	10,508		17,179	15,586	3,817	2,899		30,423	27,578	7,410	7,609	
Age (years)		53.01 ± 0.04	52.83 ± 0.04	52.88 ± 0.08	53.84 ± 0.08	<0.001	53.40 ± 0.07	53.78 ± 0.07	54.11 ± 0.14	55.69 ± 0.16	<0.001	52.79 ± 0.05	52.29 ± 0.05	52.25 ± 0.09	53.14 ± 0.09	<0.001
Education	≤Middle school	9,481 (19.92)	5,473 (12.68)	1,055 (9.40)	907 (8.63)	<0.001	1,821 (10.60)	1,170 (7.51)	215 (5.63)	170 (5.86)	<0.001	7,660 (25.18)	4,303 (15.60)	840 (11.34)	737 (9.69)	<0.001
	≤High school	27,500 (57.77)	25,001 (57.92)	6,400 (57.01)	6,082 (57.88)		9,622 (56.01)	7,922 (50.83)	1,818 (47.63)	1,326 (45.74)		17,878 (58.76)	17,079 (61.93)	4,582 (61.84)	4,756 (62.50)	
	≥College	10,621 (22.31)	12,690 (29.40)	3,772 (33.60)	3,519 (33.49)		5,736 (33.39)	6,494 (41.67)	1,784 (46.74)	1,403 (48.40)		4,885 (16.06)	6,196 (22.47)	1,988 (26.83)	2,116 (27.81)	
Household income	<2,000,000 KRW	17,701 (37.19)	12,571 (29.12)	2,823 (25.14)	2,686 (25.56)	<0.001	5,309 (30.90)	3,824 (24.53)	839 (21.98)	622 (21.46)	<0.001	12,392 (40.73)	8,747 (31.72)	1,984 (26.77)	2,064 (27.13)	<0.001
	<4,000,000 KRW	19,759 (41.51)	18,744 (43.43)	5,076 (45.21)	4,544 (43.24)		7,615 (44.33)	6,961 (44.66)	1,729 (45.30)	1,227 (42.32)		12,144 (39.92)	11,783 (42.73)	3,347 (45.17)	3,317 (43.59)	
	≥4,000,000 KRW	10,142 (21.31)	11,849 (27.45)	3,328 (29.64)	3,278 (31.20)		4,255 (24.77)	4,801 (30.80)	1,249 (32.72)	1,050 (36.22)		5,887 (19.35)	7,048 (25.56)	2,079 (28.06)	2,228 (29.28)	
Smoking	No	33,625 (70.64)	31,015 (71.85)	8,164 (72.72)	8,199 (78.03)	<0.001	4,446 (25.88)	4,360 (27.97)	1,016 (26.62)	851 (29.35)	<0.001	29,179 (95.91)	26,655 (96.65)	7,148 (96.46)	7,348 (96.57)	<0.001
	Past	7,098 (14.91)	6,883 (15.95)	1,748 (15.57)	1,495 (14.23)		6,720 (39.12)	6,529 (41.89)	1,639 (42.94)	1,390 (47.95)		378 (1.24)	354 (1.28)	109 (1.47)	105 (1.38)	
	Current	6,879 (14.45)	5,266 (12.20)	1,315 (11.71)	814 (7.75)		6,013 (35.00)	4,697 (30.14)	1,162 (30.44)	658 (22.70)		866 (2.85)	569 (2.06)	153 (2.06)	156 (2.05)	
Alcohol consumption	No	24,103 (50.63)	21,233 (49.19)	5,570 (49.61)	5,762 (54.83)	<0.001	3,506 (20.41)	3,002 (19.26)	757 (19.83)	609 (21.01)	<0.001	20,597 (67.70)	18,231 (66.11)	4,813 (64.95)	5,153 (67.72)	<0.001
	Past	1,745 (3.67)	1,530 (3.54)	412 (3.67)	415 (3.95)		1,182 (6.88)	1,039 (6.67)	288 (7.55)	256 (8.83)		563 (1.85)	491 (1.78)	124 (1.67)	159 (2.09)	
	Current	21,754 (45.70)	20,401 (47.26)	5,245 (46.72)	4,331 (41.22)		12,491 (72.71)	11,545 (74.07)	2,772 (72.62)	2,034 (70.16)		9,263 (30.45)	8,856 (32.11)	2,473 (33.37)	2,297 (30.19)	
Physical activity	No	25,300 (53.15)	19,838 (45.96)	4,547 (40.50)	3,720 (35.40)	<0.001	8,115 (47.24)	6,402 (41.08)	1,389 (36.39)	913 (31.49)	<0.001	17,185 (56.49)	13,436 (48.72)	3,158 (42.62)	2,807 (36.89)	<0.001
	Yes	22,302 (46.85)	23,326 (54.04)	6,680 (59.50)	6,788 (64.60)		9,064 (52.76)	9,184 (58.92)	2,428 (63.61)	1,986 (68.51)		13,238 (43.51)	14,142 (51.28)	4,252 (57.38)	4,802 (63.11)	
Prevalence of metabolic syndrome	Metabolic syndrome	11,006 (23.12)	8,847 (20.50)	2,240 (19.95)	1,939 (18.45)	<0.001	4,625 (26.92)	4,171 (26.76)	1,078 (28.24)	729 (25.15)	0.043	6,381 (20.97)	4,676 (16.96)	1,162 (15.68)	1,210 (15.90)	<0.001
	Abdominal obesity	12,089 (25.40)	9,910 (22.96)	2,537 (22.60)	2,098 (19.97)	<0.001	4,853 (28.25)	4,519 (28.99)	1,173 (30.73)	822 (28.35)	0.018	7,236 (23.78)	5,391 (19.55)	1,364 (18.41)	1,276 (16.77)	<0.001
	Low HDL-cholesterol	15,814 (33.22)	13,463 (31.19)	3,307 (29.46)	3,002 (28.57)	<0.001	3,929 (22.87)	3,629 (23.28)	881 (23.08)	607 (20.94)	0.053	11,885 (39.07)	9,834 (35.66)	2,426 (32.74)	2,395 (31.48)	<0.001
	Elevated triglycerides	12,486 (26.23)	10,548 (24.44)	2,673 (23.81)	2,236 (21.28)	<0.001	6,170 (35.92)	5,407 (34.69)	1,328 (34.79)	911 (31.42)	<0.001	6,316 (20.76)	5,141 (18.64)	1,345 (18.15)	1,325 (17.41)	<0.001
	Elevated blood pressure	20,739 (43.57)	17,808 (41.26)	4,626 (41.20)	4,290 (40.83)	<0.001	8,849 (51.51)	7,924 (50.84)	2,021 (52.95)	1,543 (53.23)	0.025	11,890 (39.08)	9,884 (35.84)	2,605 (35.16)	2,747 (36.10)	<0.001
	Elevated fasting blood glucose	12,038 (25.29)	10,230 (23.70)	2,654 (23.64)	2,522 (24.00)	<0.001	5,769 (33.58)	5,223 (33.51)	1,325 (34.71)	998 (34.43)	0.427	6,269 (20.61)	5,007 (18.16)	1,329 (17.94)	1,524 (20.03)	<0.001

One serving of nuts is 15 g. Values are mean ± standard error or number (%). *P*-values calculated by chi-squared test for categorical variables and ANOVA for continuous variables. Metabolic syndrome was determined based on the presence of three or more of abdominal obesity (waist circumference ≥90 cm in men and ≥85 cm in women); low HDL-cholesterol (<40 mg/dL in men and <50 mg/dL in women or medication use); elevated triglycerides (≥150 mg/dL); elevated blood pressure (systolic blood pressure ≥130 mmHg, diastolic blood pressure ≥85 mmHg, or medication use); and elevated fasting blood glucose (fasting blood glucose ≥100 mg/dL or medication use).

### 3.3 Energy and nutrient intake according to nut consumption frequency

Table 2 presents energy and nutrient intakes according to nut consumption frequency. The average daily energy intake was 1,727.0 kcal for total participants, with 1,824.3 kcal in men and 1,674.4 kcal in women. Among the total participants, men and women, total energy intake significantly increased with increasing nut consumption frequency (*p* for trends <0.001). In this study population, the proportions of energy intake from carbohydrate, fat, and protein were 72.2, 14.1, and 13.6%, respectively. Regarding the contribution of macronutrients to energy intake, among the total participants, men, and women, the proportion of energy derived from carbohydrate decreased with increasing nut consumption, whereas the contributions of protein and fat to energy intake showed an increasing trend (*p* for trend <0.001). The energy density of calcium, phosphorus, iron, sodium, potassium, vitamin A, vitamin B1, vitamin B2, niacin, vitamin C, vitamin B6, folate, dietary fiber, vitamin E, and cholesterol significantly increased with increasing nut consumption frequency among the total participants, men, and women (*p* for trends <0.05).

**Table 2:** Energy and nutrient intakes according to nut consumption frequency.

	Total					Men					Women				
	Non-consumers	<1 serving/week	1–2 servings/week	≥2 servings/week	*P* for trend	Non-consumers	<1 serving/week	1–2 servings/week	≥2 servings/week	*P* for trend	Non-consumers	<1 serving/week	1–2 servings/week	≥2 servings/week	*P* for trend
Energy (kcal/d)	1,639.56 ± 2.28	1,723.34 ± 2.43	1,878.52 ± 5.11	1,976.64 ± 6.01	<0.001	1,742.02 ± 3.76	1,822.47 ± 3.97	2,002.65 ± 8.86	2,088.00 ± 11.33	<0.001	1,581.71 ± 2.81	1,667.31 ± 3.02	1,814.58 ± 6.11	1,934.21 ± 7.03	<0.001
Carbohydrate (%kcal)	73.43 ± 0.03	72.38 ± 0.03	70.24 ± 0.06	68.25 ± 0.07	<0.001	72.67 ± 0.06	71.88 ± 0.05	69.74 ± 0.11	68.10 ± 0.13	<0.001	73.85 ± 0.04	72.67 ± 0.04	70.50 ± 0.08	68.30 ± 0.08	<0.001
Protein (%kcal)	13.33 ± 0.01	13.58 ± 0.01	14.13 ± 0.02	14.47 ± 0.02	<0.001	13.37 ± 0.02	13.58 ± 0.02	14.15 ± 0.04	14.45 ± 0.05	<0.001	13.31 ± 0.01	13.58 ± 0.01	14.12 ± 0.03	14.48 ± 0.03	<0.001
Fat (%kcal)	13.24 ± 0.02	14.03 ± 0.02	15.63 ± 0.05	17.28 ± 0.05	<0.001	13.95 ± 0.04	14.54 ± 0.04	16.11 ± 0.08	17.46 ± 0.09	<0.001	12.84 ± 0.03	13.75 ± 0.03	15.38 ± 0.06	17.22 ± 0.06	<0.001
Calcium (mg/1000 kcal/d)	238.33 ± 0.51	249.75 ± 0.52	272.62 ± 0.99	304.61 ± 1.12	<0.001	214.57 ± 0.73	223.71 ± 0.73	242.56 ± 1.45	271.74 ± 1.90	<0.001	251.75 ± 0.67	264.47 ± 0.68	288.10 ± 1.26	317.13 ± 1.34	<0.001
Phosphorus (mg/1000 kcal/d)	497.58 ± 0.45	505.51 ± 0.46	528.95 ± 0.86	557.78 ± 0.99	<0.001	484.69 ± 0.68	490.56 ± 0.68	512.49 ± 1.35	538.09 ± 1.75	<0.001	504.86 ± 0.59	513.96 ± 0.60	537.43 ± 1.10	565.28 ± 1.18	<0.001
Iron (mg/1000 kcal/d)	5.44 ± 0.01	5.68 ± 0.01	6.07 ± 0.02	6.48 ± 0.02	<0.001	5.20 ± 0.01	5.42 ± 0.01	5.83 ± 0.02	6.18 ± 0.03	<0.001	5.60 ± 0.01	5.82 ± 0.01	6.20 ± 0.02	6.60 ± 0.02	<0.001
Sodium (mg/1000 kcal/d)	1,453.84 ± 3.37	1,401.41 ± 3.25	1,434.79 ± 5.77	1,435.33 ± 6.16	0.003	1,455.95 ± 5.35	1,403.61 ± 5.19	1,444.74 ± 9.80	1,433.61 ± 12.12	0.048	1,452.65 ± 4.33	1,400.17 ± 4.16	1,429.67 ± 7.14	1,435.99 ± 7.14	0.041
Potassium (mg/1000 kcal/d)	1,237.48 ± 1.90	1,269.68 ± 1.93	1,353.86 ± 3.62	1,452.64 ± 4.04	<0.001	1,169.71 ± 2.82	1,196.11 ± 2.86	1,274.11 ± 5.64	1,353.9 ± 7.06	<0.001	1,275.75 ± 2.48	1,311.26 ± 2.52	1,394.94 ± 4.57	1,490.25 ± 4.82	<0.001
Vitamin A (R.E/1000 kcal/d)	260.94 ± 0.76	271.22 ± 0.73	294.59 ± 1.4	318.60 ± 1.64	<0.001	249.50 ± 1.16	259.42 ± 1.15	282.00 ± 2.31	302.37 ± 2.96	<0.001	267.40 ± 0.98	277.89 ± 0.94	301.08 ± 1.75	324.79 ± 1.97	<0.001
Vitamin B1 (mg/1000 kcal/d)	0.56 ± 0.00	0.57 ± 0.00	0.59 ± 0.00	0.59 ± 0.00	<0.001	0.57 ± 0.00	0.57 ± 0.00	0.59 ± 0.00	0.59 ± 0.00	<0.001	0.60 ± 0.00	0.57 ± 0.00	0.58 ± 0.00	0.60 ± 0.00	<0.001
Vitamin B2 (mg/1000 kcal/d)	0.49 ± 0.00	0.51 ± 0.00	0.54 ± 0.00	0.58 ± 0.00	<0.001	0.48 ± 0.00	0.49 ± 0.00	0.52 ± 0.00	0.55 ± 0.00	<0.001	0.50 ± 0.00	0.52 ± 0.00	0.55 ± 0.00	0.59 ± 0.00	<0.001
Niacin (mg/1000 kcal/d)	8.11 ± 0.01	8.23 ± 0.01	8.66 ± 0.02	9.20 ± 0.02	<0.001	8.14 ± 0.01	8.24 ± 0.01	8.69 ± 0.03	9.16 ± 0.03	<0.001	8.08 ± 0.01	8.23 ± 0.01	8.65 ± 0.02	9.22 ± 0.02	<0.001
Vitamin C (mg/1000 kcal/d)	57.44 ± 0.15	59.93 ± 0.15	64.9 ± 0.29	69.68 ± 0.31	<0.001	49.88 ± 0.20	52.53 ± 0.21	56.57 ± 0.43	60.43 ± 0.52	<0.001	61.71 ± 0.19	64.12 ± 0.19	69.20 ± 0.37	73.21 ± 0.38	<0.001
Vitamin B6 (mg/1000 kcal/d)	0.89 ± 0.00	0.9 ± 0.00	0.94 ± 0.00	0.97 ± 0.00	<0.001	0.86 ± 0.00	0.87 ± 0.00	0.91 ± 0.00	0.93 ± 0.00	<0.001	0.90 ± 0.00	0.92 ± 0.00	0.95 ± 0.00	0.98 ± 0.00	<0.001
Folate (μg/1000 kcal/d)	118.59 ± 0.24	122.33 ± 0.24	131.82 ± 0.45	145.56 ± 0.52	<0.001	110.56 ± 0.36	114.15 ± 0.37	122.67 ± 0.71	134.64 ± 0.93	<0.001	123.13 ± 0.31	126.96 ± 0.31	136.53 ± 0.56	149.73 ± 0.62	<0.001
Dietary fiber (g/1000 kcal/d)	3.19 ± 0.01	3.24 ± 0.01	3.43 ± 0.01	3.74 ± 0.01	<0.001	2.98 ± 0.01	3.03 ± 0.01	3.21 ± 0.02	3.48 ± 0.02	<0.001	3.31 ± 0.01	3.36 ± 0.01	3.55 ± 0.01	3.84 ± 0.01	<0.001
Vitamin E (mg/1000 kcal/d)	4.21 ± 0.01	4.53 ± 0.01	5.06 ± 0.01	5.81 ± 0.02	<0.001	4.06 ± 0.01	4.38 ± 0.01	4.90 ± 0.02	5.55 ± 0.03	<0.001	4.29 ± 0.01	4.62 ± 0.01	5.14 ± 0.02	5.91 ± 0.02	<0.001
Cholesterol (mg/1000 kcal/d)	57.44 ± 0.15	59.93 ± 0.15	64.9 ± 0.29	69.68 ± 0.31	<0.001	49.88 ± 0.20	52.53 ± 0.21	56.57 ± 0.43	60.43 ± 0.52	<0.001	61.71 ± 0.19	64.1 ± 0.19	69.2 ± 0.37	73.2 ± 0.38	<0.001

One serving of nuts is 15 g. Values are mean ± standard error. Covariates considered potential confounding variables included age (continuous), body mass index (BMI, continuous), educational level (middle school or less, high school or less, or college or more), household income (<2,000,000, <4,000,000, or ≥4,000,000 KRW), smoking status (current, past, or never), alcohol consumption (current, past, or never), and physical activity (yes or no). In analyses of the total participants, sex was included as a covariate.

### 3.4 Anthropometric and biochemical variables according to nut consumption frequency

The anthropometric and biochemical variables according to nut consumption frequency are listed in Table 3. Among all participants, the highest consumption group had lower BMI, waist circumference, SBP, DBP, and triglycerides but higher levels of total cholesterol, HDL-, and LDL-cholesterol than non-consumers (*p* for trends <0.05). Fasting blood glucose levels were not associated with nut consumption. In men, higher nut consumption was inversely associated with waist circumference, blood pressure, and triglycerides but positively associated with levels of total cholesterol, HDL-cholesterol, and LDL-cholesterol (*p* for trends <0.05). Among women, individuals with a higher nut consumption frequency had a smaller waist circumference, lower BMI, DBP, and triglycerides but higher levels of total cholesterol, HDL-cholesterol, and LDL-cholesterol than non-consumers (*p* for trends <0.05).

**Table 3:** Anthropometric and biochemical variables according to nut consumption frequency.

	Total					Men					Women				
	Non-consumers	<1 serving/week	1–2 servings/week	≥2 servings/week	*P* for trend	Non-consumers	<1 serving/week	1–2 servings/week	≥2 servings/week	*P* for trend	Non-consumers	<1 serving/week	1–2 servings/week	≥2 servings/week	*P* for trend
Body mass index (kg/m^2^)	23.95 ± 0.01	23.83 ± 0.01	23.81 ± 0.03	23.64 ± 0.03	<0.001	24.27 ± 0.02	24.40 ± 0.02	24.51 ± 0.05	24.43 ± 0.05	<0.001	23.76 ± 0.02	23.50 ± 0.02	23.44 ± 0.03	23.35 ± 0.03	<0.001
Waist circumference (cm)	81.27 ± 0.04	80.72 ± 0.04	80.39 ± 0.08	79.59 ± 0.08	<0.001	85.40 ± 0.06	85.68 ± 0.06	85.78 ± 0.12	85.48 ± 0.14	<0.001	78.94 ± 0.05	77.91 ± 0.05	77.62 ± 0.09	77.34 ± 0.09	<0.001
Systolic blood pressure (mmHg)	122.52 ± 0.07	121.94 ± 0.07	121.75 ± 0.14	121.35 ± 0.14	0.011	125.21 ± 0.11	125.03 ± 0.11	125.06 ± 0.22	125.00 ± 0.26	0.041	121.01 ± 0.09	120.19 ± 0.09	120.05 ± 0.17	119.96 ± 0.17	0.160
Diastolic blood pressure (mmHg)	76.11 ± 0.05	75.64 ± 0.05	75.43 ± 0.09	75.24 ± 0.10	<0.001	78.39 ± 0.07	78.13 ± 0.08	78.11 ± 0.15	78.03 ± 0.18	0.003	74.82 ± 0.06	74.24 ± 0.06	74.05 ± 0.11	74.17 ± 0.11	0.005
Total cholesterol (mg/dL)	195.70 ± 0.16	196.49 ± 0.17	197.84 ± 0.33	198.85 ± 0.35	<0.001	191.65 ± 0.26	192.62 ± 0.28	193.15 ± 0.55	192.43 ± 0.65	0.023	197.99 ± 0.20	198.68 ± 0.21	200.26 ± 0.41	201.30 ± 0.41	<0.001
HDL-cholesterol (mg/dL)	52.83 ± 0.06	53.64 ± 0.06	54.62 ± 0.12	56.32 ± 0.13	<0.001	49.24 ± 0.09	49.30 ± 0.09	49.49 ± 0.19	50.57 ± 0.22	<0.001	54.86 ± 0.07	56.09 ± 0.08	57.26 ± 0.15	58.51 ± 0.16	<0.001
LDL-cholesterol (mg/dL)	118.47 ± 0.15	119.14 ± 0.15	119.79 ± 0.3	120.06 ± 0.31	0.002	114.39 ± 0.24	115.86 ± 0.25	116.21 ± 0.51	115.80 ± 0.59	0.007	120.77 ± 0.19	120.99 ± 0.19	121.63 ± 0.37	121.69 ± 0.37	0.047
Triglycerides (mg/dL)	122.02 ± 0.31	118.58 ± 0.32	117.17 ± 0.62	112.37 ± 0.61	<0.001	115.25 ± 0.16	140.13 ± 0.57	137.29 ± 0.58	137.23 ± 1.19	<0.001	111.79 ± 0.35	108.01 ± 0.35	106.83 ± 0.67	105.53 ± 0.66	<0.001
Fasting blood glucose (mg/dL)	95.22 ± 0.10	94.64 ± 0.09	94.56 ± 0.20	94.35 ± 0.18	0.271	98.58 ± 0.18	98.64 ± 0.18	99.07 ± 0.39	98.62 ± 0.39	0.781	93.32 ± 0.12	92.38 ± 0.11	92.24 ± 0.22	92.73 ± 0.19	0.259

One serving of nuts is 15 g. Values are mean ± standard error. Covariates considered potential confounding variables included energy intake (continuous), age (continuous), educational level (middle school or less, high school or less, or college or more), household income (<2,000,000, <4,000,000, or ≥4,000,000 KRW), smoking status (current, past, or never), alcohol consumption (current, past, or never), physical activity (yes or no), and body mass index (BMI; continuous except for “BMI” variable). In analyses of the total participants, sex was included as a covariate.

### 3.5 Association of nut consumption with metabolic syndrome and its components

**Figure 2:**
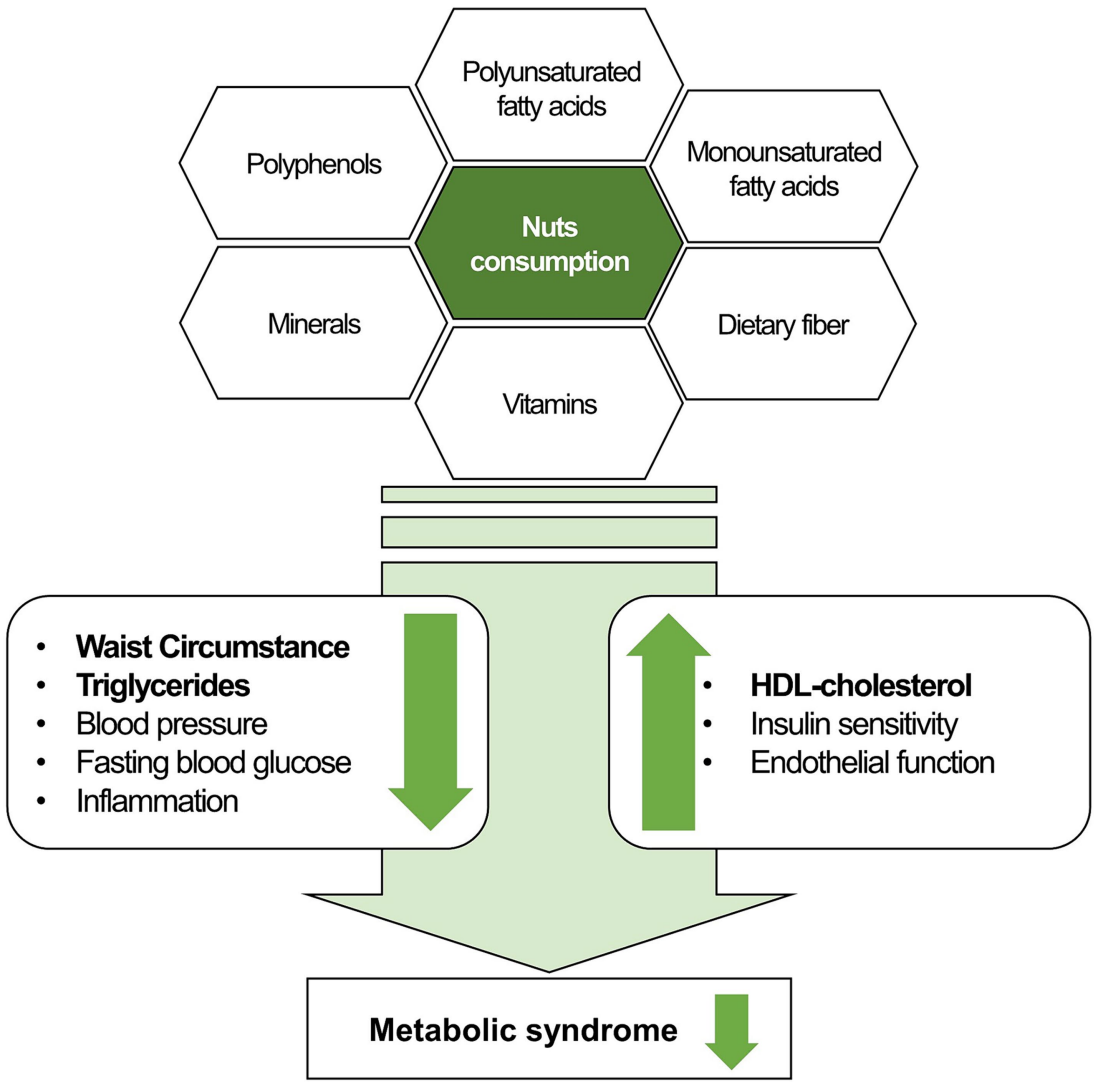
The role of nut consumption in prevention and management of metabolic syndrome. Bold within the figure indicates significant results from this study.

Table 4 presents the association between nut consumption frequency and metabolic syndrome and its components. After adjusting for covariates, the ORs for metabolic syndrome were significantly lower in the group consuming ≥2 servings/week than the non-consumers group among the total participants (OR = 0.85, 95% CI = 0.80 to 0.91, *p* for trend <0.001), men (OR = 0.86, 95% CI = 0.77 to 0.95, *p* for trend = 0.028), and women (OR = 0.86, 95% CI = 0.80 to 0.93, *p* for trend <0.001). In both men and women, the group with the highest nut consumption frequency (≥2 servings/week) showed significantly lower ORs for abdominal obesity, low HDL-cholesterol, and elevated triglycerides than non-consumers. Nut consumption was not significantly associated with elevated blood pressure or fasting blood glucose. The overall concept on the role of nut intake in prevention and management of metabolic syndrome is summarized in Figure 2.

**Table 4:** Associations of nut consumption with metabolic syndrome and its components.

	Total					Men					Women				
	Non-consumers	<1 serving/week	1–2 servings/week	≥2 servings/week	*P* for trend	Non-consumers	<1 serving/week	1–2 servings/week	≥2 servings/week	*P* for trend	Non-consumers	<1 serving/week	1–2 servings/week	≥2 servings/week	*P* for trend
Metabolic syndrome	1.00 (ref)	0.92 (0.89,0.95)	0.91 (0.86,0.96)	0.85 (0.80,0.91)	<0.001	1.00 (ref)	0.97 (0.92,1.02)	1.01 (0.93,1.11)	0.86 (0.77,0.95)	0.028	1.00 (ref)	0.89 (0.85,0.93)	0.85 (0.79,0.92)	0.86 (0.80,0.93)	<0.001
Abdominal obesity	1.00 (ref)	0.93 (0.89,0.97)	0.90 (0.84,0.96)	0.77 (0.72,0.83)	<0.001	1.00 (ref)	0.98 (0.92,1.05)	0.98 (0.89,1.09)	0.86 (0.76,0.97)	0.044	1.00 (ref)	0.89 (0.85,0.94)	0.84 (0.77,0.92)	0.73 (0.67,0.80)	<0.001
Low HDL-cholesterol	1.00 (ref)	0.96 (0.93,0.99)	0.88 (0.84,0.92)	0.80 (0.76,0.84)	<0.001	1.00 (ref)	1.03 (0.98,1.09)	1.00 (0.92,1.09)	0.89 (0.80,0.98)	0.149	1.00 (ref)	0.93 (0.89,0.96)	0.83 (0.79,0.88)	0.77 (0.73,0.82)	<0.001
Elevated triglycerides	1.00 (ref)	0.95 (0.92,0.98)	0.94 (0.90,0.99)	0.88 (0.83,0.93)	<0.001	1.00 (ref)	0.96 (0.91,1.00)	0.95 (0.88,1.03)	0.88 (0.80,0.96)	0.004	1.00 (ref)	0.95 (0.91,0.99)	0.95 (0.89,1.02)	0.89 (0.83,0.95)	0.001
Elevated blood pressure	1.00 (ref)	0.95 (0.93,0.98)	0.98 (0.94,1.03)	0.98 (0.93,1.02)	0.112	1.00 (ref)	0.94 (0.90,0.99)	1.03 (0.96,1.11)	1.00 (0.92,1.09)	0.932	1.00 (ref)	0.96 (0.93,1.00)	0.96 (0.91,1.02)	0.97 (0.92,1.03)	0.140
Elevated fasting blood glucose	1.00 (ref)	0.96 (0.93,0.99)	0.99 (0.94,1.04)	1.05 (1.00,1.11)	0.398	1.00 (ref)	0.99 (0.94,1.04)	1.04 (0.96,1.12)	1.00 (0.91,1.09)	0.733	1.00 (ref)	0.94 (0.90,0.98)	0.96 (0.89,1.02)	1.09 (1.02,1.16)	0.264

One serving of nuts is 15 g. Values are odds ratio (95% confidence interval). Covariates considered potential confounding variables included energy intake (continuous), age (continuous), body mass index (BMI, continuous), educational level (middle school or less, high school or less, or college or more), household income (<2,000,000, <4,000,000, or ≥4,000,000), smoking status (current, past, or never), alcohol consumption (current, past, or never), and physical activity (yes or no). In analyses of the total participants, sex was included as a covariate. Metabolic syndrome was determined based on the presence of three or more of abdominal obesity (waist circumference ≥90 cm in men and ≥85 cm in women); low HDL-cholesterol (<40 mg/dL in men and <50 mg/dL in women or medication use); elevated triglycerides (triglycerides ≥ 150 mg/dL); elevated blood pressure (systolic blood pressure ≥ 130 mmHg or diastolic blood pressure ≥ 85 mmHg or medication use); and elevated fasting blood glucose (fasting blood glucose ≥ 100 mg/dL or medication use).

## 4 Discussion

### 4.1 Main findings of this study

This study utilized a large-scale dataset from the KoGES-HEXA to investigate the association of nut consumption frequency with metabolic syndrome and its components among Korean adults. The findings of this study indicated that an increased frequency of nut consumption was associated with a decreased prevalence of metabolic syndrome in Korean adults. Among the components of metabolic syndrome, inverse associations of nut consumption with abdominal obesity, low HDL-cholesterol levels, and elevated triglycerides levels were observed in both men and women. However, no significant association was observed between nut consumption and increased blood pressure or fasting blood glucose.

This study aligns with previous meta-analyses that revealed a decreased risk of metabolic syndrome with higher nut consumption. A meta-analysis of prospective cohort studies examined the relationship between nut consumption and the risk of metabolic syndrome and revealed that for each additional serving per week (30 g) of nuts, the risk of metabolic syndrome decreased by 4% (relative risk (RR) = 0.96, 95% CI = 0.92 to 0.99) (11). Another meta-analysis comparing high and low consumption of nuts reported an RR of 0.84 (95% CI = 0.76 to 0.92) for metabolic syndrome in the high-consumption group (28). Our finding was also consistent with a previous cohort study of Korean adults that used the Ansan–Ansung cohort study data, which discovered an inverse relationship between consuming >15 g of nuts/week and the incidence of metabolic syndrome (RR = 0.72, 95% CI = 0.56 to 0.93 in men, RR = 0.57, 95% CI = 0.41 to 0.79 in women) (14). Korean adults generally consume less nuts than individuals in the Mediterranean or Western regions; however, our research findings revealed a significant inverse association between nut consumption and risk reduction of metabolic syndrome and its components. This finding suggests that, even in populations with lower nut consumption, nuts may offer protection against metabolic syndrome because they contain beneficial nutrients. Similar to findings from previous Western studies, our results indicate that nut consumption among Koreans could have positive implications for improving cardiometabolic diseases such as CVD and metabolic syndrome.

### 4.2 Underlying mechanism of the association between nut consumption and metabolic syndrome

The inverse relationship between higher nut consumption and reduced risk of metabolic syndrome may be because nuts are rich in nutrients that are beneficial in preventing metabolic syndrome. Specifically, nuts have a high polyunsaturated fatty acid content, which reduces plasma triglycerides levels, thereby contributing to the amelioration of risk factors associated with metabolic syndrome (29). Dietary fiber in nuts is known to influence the gut microbiota; the fermentation of dietary fiber by these microbiota generates short-chain fatty acids as byproducts, lowering blood pressure (30). Furthermore, antioxidants such as vitamin E and carotenoids in nuts might reduce chronic inflammation and lower the risk of related diseases, thus aiding in the prevention of CVD (31, 32) (Figure 2). Therefore, dietary guidelines from the American Heart Association, DASH, and Mediterranean diet patterns consistently recommend regular moderate consumption of nuts (8, 9, 33).

Moreover, a study on a Mediterranean population with high CVD risk suggested that nut consumption is an important indicator of diet quality (34). A study using 2005–2010 National Health and Nutrition Examination Survey (NHANES) data also reported that tree nut consumption is associated with better nutrient adequacy and diet quality in adults in the US (35). In this study, the nutrient density of vitamins and minerals increased with increased nut consumption frequency, suggesting that nut consumption might be related to higher nutrient quality in the diets of Korean adults. Consequently, a higher diet quality could contribute to the prevention and management of metabolic syndrome.

### 4.3 Impact of nut consumption on lipid profiles and abdominal obesity

In the current study, among the metabolic syndrome components, higher nut consumption was inversely associated with abdominal obesity and dyslipidemia in both men and women. Compared with those who did not consume nuts, men and women who consumed nuts more than twice a week exhibited reduced ORs for low HDL-cholesterol and elevated triglycerides. These findings are consistent with a systematic review targeting healthy adults with overweight/obesity, which revealed that nut consumption significantly decreased triglycerides levels (weighted mean difference = −13.19 mg/dL, 95% CI = −25.90 to −0.48) (13). Additionally, a cross-sectional study involving Iranian men aged 60–74 years revealed that the highest tertile group of nut consumption resulted in 2.24 times higher HDL-cholesterol levels compared with the lowest tertile group (36). Participants with metabolic syndrome and BMI ≥ 23 kg/m^2^ were included in a Korean randomized, parallel, controlled study conducted over 6 weeks and revealed that women who consumed 30 g of nuts per day had significantly improved total cholesterol and non-HDL-cholesterol levels than controls (15). This inverse association between nuts and dyslipidemia could be attributed to the high content of polyunsaturated and monounsaturated fatty acids in nuts, positively affecting lipid metabolism (37).

Nuts have a high-fat content, resulting in high energy density (38). However, this study discovered that increased nut consumption among both men and women resulted in a tendency for decreased prevalence of abdominal obesity. A meta-analysis revealed that an increase of one serving per week in nut consumption was associated with a 3% reduction in the risk of overweight/obesity (RR = 0.97, 95% CI = 0.95 to 0.98) (11). Another meta-analysis of prospective cohort studies reported that nut consumption was associated with a reduction in the risk of overweight/obesity, with an RR of 0.93 (95% CI = 0.88–0.98). Moreover, replacing carbohydrate snacks with nuts during the intervention period of 16 weeks decreased waist circumference in US women, with a mean difference of −2.20 cm, and showed a trend toward reduced visceral fat, with a change of −5.27 ± 13.05 cm^2^ (39). Nut consumption may promote satiety because of the high protein and dietary fiber content of nuts, thereby suppressing hunger and appetite and reducing energy consumption and abdominal obesity (40, 41). The prebiotic effects of nuts on the gut microbiome may contribute to weight control (11).

### 4.4 Impact of nut consumption on blood pressure and fasting blood glucose

In this study, nut consumption was not significantly associated with blood pressure or fasting blood glucose levels. A cohort study in China involving participants aged ≥50 years revealed no relationship between nut consumption and the Framingham score, SBP, or DBP (42). The Seguimiento Universidad de Navarra Project, a dynamic cohort study consisting exclusively of Spanish university graduates in Spain, revealed no association between nut consumption and the risk of hypertension (RR = 0.77, 95% CI = 0.46–1.30) (43). However, a meta-analysis reported an inverse correlation between nut consumption and the risk of hypertension (RR = 0.84, 95% CI = 0.76–0.93) (44). Unsaturated fatty acids in nuts exert hypotensive effects (45). Additionally, dietary fiber, protein, arginine, and minerals such as magnesium, potassium, and calcium in nuts are potentially beneficial for hypertension (10, 32, 46).

Previous findings on nut consumption and glucose control have been inconsistent. A meta-analysis of randomized controlled trials reported that nut consumption had a favorable effect on fasting insulin and HOMA-IR but no significant effect on fasting blood glucose (47). Among healthy adults who were overweight/obese in several randomized controlled trials, nuts did not affect glycemic measures, including fasting blood glucose, fasting insulin, and HOMA-IR (13). Similarly, a study of obese adults with an average age of 40.78 years in Iran revealed that a healthful plant-based diet which included nuts showed no correlation with hyperglycemia (48). This study focused solely on fasting blood glucose levels and discovered no significant association with nut consumption. Further research is needed to completely understand the relationship between nut consumption, hypertension, and type 2 diabetes.

### 4.5 Variability in nut consumption and its impact on metabolic syndrome

Inconsistent results regarding the relationship between nut consumption and metabolic syndrome and its components across studies can be attributed to multiple factors. First, the frequency of nut consumption differed across the study populations. The 2012 NHANES revealed that one-third of US adults consumed at least five servings of nuts and seeds per week (1 oz. or 28.3 g) (49). Similarly, among nut consumers in Australia, the average nut intake was 11.75 g per day (50). In the Korean adults in this study, a relatively lower consumption of nuts was observed than in Western populations. Second, the types of nuts consumed by the study populations might differentially influence health outcomes due to the different nutrient and functional compound contents. For example, macadamia nuts and pecans have the highest caloric content per 100 g; walnuts contain the most polyunsaturated fatty acids; and almonds and pistachios are protein-rich. Furthermore, pistachios and cashews are notable for their high mineral contents, such as potassium and iron (51). Third, the preparation and processing of nuts, such as salting or roasting, may alter their nutritional content and health effects compared to those of raw nuts. These factors, including the amount, type, preparation, and processing of nuts consumed by study populations, might collectively influence the relationship between nut consumption and metabolic syndrome; thus, these factors need to be considered when collecting dietary data and analyses.

### 4.6 Limitations and strengths

This cross-sectional study had limitations in establishing causality between nut consumption and metabolic syndrome. It grouped peanuts, almonds, and pine nuts rather than analyzing them individually and did not consider other types of nuts in their association with metabolic syndrome. Additionally, the salt content and preparation methods (e.g., roasting) of the nuts consumed by the participants were not examined. Although covariates that could affect nut consumption and metabolic syndrome were adjusted, other potential confounding variables remained. For example, details regarding the exact time or frequency of physical activity were not considered. Additionally, this study could not exclude participants who followed a special diet under certain conditions.

Despite these limitations, the findings of this study provide insights into the health effects of nut consumption on metabolic syndrome based on a large-scale dataset of Korean adults. Additionally, this study is significant to understand underlying mechanism of nut intake and metabolic syndrome because it examined the association of nut consumption with metabolic syndrome components. Future studies with detailed specifications of nut varieties and nut processing conditions are necessary to further elaborate on the results of this study.

### 4.7 Conclusion

In Korean adults, increased frequency of nut consumption was associated with protective effects against metabolic syndrome, abdominal obesity, reduced HDL-cholesterol, and elevated triglycerides. Consistent with previous meta-analyses that predominantly involved Western populations, this study suggests that nut consumption could positively impact the reduction in metabolic syndrome and CVD risk among Koreans. Therefore, to prevent metabolic syndrome and its components, it is recommended that frequent nut consumption be incorporated into the diet.

The findings of this study may contribute to the development of dietary guidelines and public health policies for CVD prevention in this population. This study demonstrated that improving dietary habits can lower the risk of metabolic syndrome and CVD, supporting the inclusion of a balanced diet with nuts in clinical practice for the prevention and management of these diseases. Emphasizing non-pharmacological strategies, these findings encourage metabolic physicians, endocrinologists, and diabetologists to adopt these dietary recommendations. Utilizing these guidelines can facilitate early detection and targeted interventions at the primary prevention stage, essential for addressing factors influencing adult metabolism and preventing adverse cardiometabolic disorders. Further cohort and clinical studies are required to explore the complex effects of the quantity and type of nuts consumed on development of metabolic diseases.

## Data availability statement

Publicly available datasets were analyzed in this study. This data can be found here: http://nih.go.kr/ko/main/contents.do?menuNo=300563.

## Ethics statement

The studies involving humans were approved by the institutional review board of Hannam University (2023-E-01-09-0625). The studies were conducted in accordance with the local legislation and institutional requirements. The participants provided their written informed consent to participate in this study.

## Author contributions

HS: Formal analysis, Methodology, Writing – original draft, Writing – review & editing. SS: Conceptualization, Project administration, Writing – original draft, Writing – review & editing.

## References

[1] National Heart, Lung, and Blood Institute. What is metabolic syndrome? Curr Hypertens Rep. (2022) [updated May 18, 2022]. Available at: https://www.nhlbi.nih.gov/health/metabolic-syndrome.

[2] Galassi A Reynolds K He J. Metabolic syndrome and risk of cardiovascular disease: a meta-analysis. Am J Med. (2006) 119:812–9. doi: 10.1016/j.amjmed.2006.02.031 17000207

[3] National Cholesterol Education Program (NCEP) Expert Panel on Detection, Evaluation, and Treatment of High Blood Cholesterol in Adults (Adult Treatment Panel III). Third Report of the National Cholesterol Education Program (NCEP) Expert Panel on Detection, Evaluation, and Treatment of High Blood Cholesterol in Adults (Adult Treatment Panel III) final report. Circulation. (2002) 106:3143–3421. doi: 10.1161/circ.106.25.3143 12485966

[4] Vaduganathan M Mensah GA Turco JV Fuster V Roth GA. The global burden of cardiovascular diseases and risk. J Am Coll Cardiol. (2022) 80:2361–71. doi: 10.1016/j.jacc.2022.11.005 36368511

[5] Nikniaz L Mahmudiono T Jasim SA Vajdi M Thangavelu L Farhangi MA. Nutrient pattern analysis of mineral based, simple sugar based, and fat based diets and risk of metabolic syndrome: a comparative nutrient panel. BMC Endocr Disord. (2022) 22:51. doi: 10.1186/s12902-022-00963-2, PMID: 35232417 PMC8889682

[6] Vajdi M Farhangi MA Nikniaz L. Diet-derived nutrient patterns and components of metabolic syndrome: a cross-sectional community-based study. BMC Endocr Disord. (2020) 20:69. doi: 10.1186/s12902-020-0547-0, PMID: 32429966 PMC7236137

[7] Vajdi M Karimi A Farhangi MA Ardekani AM. The association between healthy lifestyle score and risk of metabolic syndrome in Iranian adults: a cross-sectional study. BMC Endocr Disord. (2023) 23:16. doi: 10.1186/s12902-023-01270-0, PMID: 36647030 PMC9843981

[8] Appel LJ Moore TJ Obarzanek E Vollmer WM Svetkey LP Sacks FM. A clinical trial of the effects of dietary patterns on blood pressure. N Engl J Med. (1997) 336:1117–24. doi: 10.1056/nejm199704173361601 9099655

[9] Martínez-González MA Gea A Ruiz-Canela M. The Mediterranean diet and cardiovascular health. Circ Res. (2019) 124:779–98. doi: 10.1161/CIRCRESAHA.118.313348 30817261

[10] De Souza RGM Schincaglia RM Pimentel GD Mota JF. Nuts and human health outcomes: a systematic review. Nutrients. (2017) 9:1311. doi: 10.3390/nu9121311 29207471 PMC5748761

[11] Li H Li X Yuan S Jin Y Lu J. Nut consumption and risk of metabolic syndrome and overweight/obesity: a meta-analysis of prospective cohort studies and randomized trials. Nutr Metab. (2018) 15:46. doi: 10.1186/s12986-018-0282-y, PMID: 29977320 PMC6013998

[12] Blanco Mejia S Kendall CW Viguiliouk E Augustin LS Ha V Cozma AI. Effect of tree nuts on metabolic syndrome criteria: a systematic review and meta-analysis of randomized controlled trials. BMJ Open. (2014) 4:e004660. doi: 10.1136/bmjopen-2013-004660, PMID: 25074070 PMC4120343

[13] Eslami O Khorramrouz F Sohouli M Bagheri N Shidfar F Fernandez ML. Effect of nuts on components of metabolic syndrome in healthy adults with overweight/obesity: a systematic review and meta-analysis. Nutr Metab Cardiovasc Dis. (2022) 32:2459–69. doi: 10.1016/j.numecd.2022.07.015, PMID: 36058762

[14] Jung JY Park SK Oh C-M Choi J-M Ryoo J-H Kim J. The association between metabolic syndrome and peanuts, pine nuts, almonds consumption: the Ansan and Ansung study. Endocrine. (2019) 65:270–7. doi: 10.1007/s12020-019-01980-3, PMID: 31243651

[15] Lee YJ Nam GE Seo JA Yoon T Seo I Lee JH. Nut consumption has favorable effects on lipid profiles of Korean women with metabolic syndrome. Nutr Res. (2014) 34:814–20. doi: 10.1016/j.nutres.2014.08.011, PMID: 25238912

[16] Lobene AJ. The increasing prevalence of metabolic syndrome in Korea: a multifarious disease with a multifactorial etiology. JACC Asia. (2023) 3:503–5. doi: 10.1016/j.jacasi.2023.05.004, PMID: 37396420 PMC10308152

[17] Statistics Korea. Causes of death statistics in 2021. Daejeon: Statistics Korea (2022).

[18] Kim Y Han B-G KoGES Consortium. Cohort profile: the Korean genome and epidemiology study (KoGES) consortium. Int J Epidemiol. (2017) 46:e20. doi: 10.1093/ije/dyv316, PMID: 27085081 PMC5837648

[19] National Institute of Health. The Korean genome and epidemiology study (KoGES). Osong: National Institute of Health (2022).

[20] Kwon YS Ryu J Yang Y Park YK Kim S. Trends and dietary assessment according to fruit and vegetable intake in Korean elderly people: analysis based on the Korea National Health and nutrition examination survey 1998, 2008, and 2018. Food Secur. (2020) 9:1712. doi: 10.3390/foods9111712, PMID: 33266368 PMC7700258

[21] Sung H Park JM Oh SU Ha K Joung H. Consumption of ultra-processed foods increases the likelihood of having obesity in Korean women. Nutrients. (2021) 13:698. doi: 10.3390/nu13020698, PMID: 33671557 PMC7926298

[22] Jo U Park K. Carbohydrate-based diet may increase the risk of cardiovascular disease: a pooled analysis of two prospective cohort studies. Clin Nutr. (2023) 42:1301–7. doi: 10.1016/j.clnu.2023.06.013, PMID: 37385184

[23] Ahn Y Kwon E Shim JE Park MK Joo Y Kimm K. Validation and reproducibility of food frequency questionnaire for Korean genome epidemiologic study. Eur J Clin Nutr. (2007) 61:1435–41. doi: 10.1038/sj.ejcn.1602657, PMID: 17299477

[24] Kang HT Kim SY Kim J Kim J Kim J Park HA. Clinical practice guideline of prevention and treatment for metabolic syndrome. Korean J Fam Pract. (2015) 5:375–420.

[25] Huh JH Kang DR Kim JY Koh KK. Metabolic syndrome fact sheet 2021: executive report. Cardiometab Syndr J. (2021) 1:125–34. doi: 10.51789/cmsj.2021.1.e15

[26] Jung H Shin J Lim K Shin S. Edible mushroom intake and risk of all-cause and cause-specific mortality: results from the Korean genome and epidemiology study (KoGES) cohort. Food Funct. (2023) 14:8829–37. doi: 10.1039/D3FO00996C 37682230

[27] Health Examinees Study Group. The health examinees (HEXA) study: rationale, study design and baseline characteristics. Asian Pac J Cancer Prev. (2015) 16:1591–7. doi: 10.7314/apjcp.2015.16.4.1591, PMID: 25743837

[28] Zhang Y Zhang D-Z. Relationship between nut consumption and metabolic syndrome: a meta-analysis of observational studies. J Am Coll Nutr. (2019) 38:499–505. doi: 10.1080/07315724.2018.1561341, PMID: 30716015

[29] Bornfeldt KE. Triglyceride lowering by omega-3 fatty acids: a mechanism mediated by n-acyl taurines. J Clin Invest. (2021) 131:e147558. doi: 10.1172/JCI147558, PMID: 33720044 PMC7954591

[30] Xu C Marques FZ. How dietary fibre, acting via the gut microbiome, lowers blood pressure. Curr Hypertens Rep. (2022) 24:509–21. doi: 10.1007/s11906-022-01216-2, PMID: 35838884 PMC9568477

[31] Jackson CL Hu FB. Long-term associations of nut consumption with body weight and obesity. Am J Clin Nutr. (2014) 100:408S–11S. doi: 10.3945/ajcn.113.071332, PMID: 24898229 PMC4144111

[32] de Souza RJ Dehghan M Mente A Bangdiwala SI Ahmed SH Alhabib KF. Association of nut intake with risk factors, cardiovascular disease, and mortality in 16 countries from 5 continents: analysis from the PURE study. Am J Clin Nutr. (2020) 112:208–19. doi: 10.1093/ajcn/nqaa108, PMID: 32433740

[33] American Heart Association. The American Heart Association diet and lifestyle recommendations 2023. Available at: https://www.heart.org/en/healthy-living/healthy-eating/eat-smart/nutrition-basics/aha-diet-and-lifestyle-recommendations.

[34] Bibiloni MM Julibert A Bouzas C Martínez-González MA Corella D Salas-Salvadó J. Nut consumptions as a marker of higher diet quality in a Mediterranean population at high cardiovascular risk. Nutrients. (2019) 11:754. doi: 10.3390/nu11040754, PMID: 30935087 PMC6521169

[35] O'Neil CE Nicklas TA Fulgoni VL. Tree nut consumption is associated with better nutrient adequacy and diet quality in adults: National Health and nutrition examination survey 2005-2010. Nutrients. (2015) 7:595–607. doi: 10.3390/nu7010595, PMID: 25599274 PMC4303856

[36] Askari M Daneshzad E Jafari A Bellissimo N Azadbakht L. Association of nut and legume consumption with Framingham 10 year risk of general cardiovascular disease in older adult men: a cross-sectional study. Clin Nutr ESPEN. (2021) 42:373–80. doi: 10.1016/j.clnesp.2020.12.024, PMID: 33745608

[37] Tapsell L Batterham M Tan S-Y Warensjö E. The effect of a calorie-controlled diet containing walnuts on substrate oxidation during 8-hours in a room calorimeter. J Am Coll Nutr. (2009) 28:611–7. doi: 10.1080/07315724.2009.10719793, PMID: 20439557

[38] Nishi SK Viguiliouk E Blanco Mejia S Kendall CWC Bazinet RP Hanley AJ. Are fatty nuts a weighty concern? A systematic review and meta-analysis and dose–response meta-regression of prospective cohorts and randomized controlled trials. Obes Rev. (2021) 22:e13330. doi: 10.1111/obr.13330, PMID: 34494363 PMC9285885

[39] Sumislawski K Widmer A Suro RR Robles ME Lillegard K Olson D. Consumption of tree nuts as snacks reduces metabolic syndrome risk in young adults: a randomized trial. Nutrients. (2023) 15:5051. doi: 10.3390/nu15245051, PMID: 38140310 PMC10745921

[40] Tan SY Mattes RD. Appetitive, dietary, and health effects of almonds consumed with meals or as snacks: a randomized, controlled trial. Eur J Clin Nutr. (2013) 67:1205–14. doi: 10.1038/ejcn.2013.184, PMID: 24084509 PMC3898316

[41] Guarneiri LL Cooper JA. Intake of nuts or nut products does not lead to weight gain, independent of dietary substitution instructions: a systematic review and meta-analysis of randomized trials. Adv Nutr. (2021) 12:384–401. doi: 10.1093/advances/nmaa113, PMID: 32945861 PMC8009751

[42] Sun Y Jiang CQ Cheng KK Zhang WS Leung GM Lam TH. Nut consumption and cardiovascular risk in older Chinese: the Guangzhou biobank cohort study. PLoS One. (2015) 10:e0137178. doi: 10.1371/journal.pone.0137178, PMID: 26332759 PMC4558011

[43] Martínez-Lapiscina EH Pimenta AM Beunza JJ Bes-Rastrollo M Martínez JA Martínez-González MA. Nut consumption and incidence of hypertension: the SUN prospective cohort. Nutr Metab Cardiovasc Dis. (2010) 20:359–65. doi: 10.1016/j.numecd.2009.04.013, PMID: 19683907

[44] Guo K Jiang Y Zhou Z Li Y. Retracted: nut consumption with risk of hypertension and type 2 diabetes mellitus: a meta-analysis of prospective cohort studies. Eur J Prev Cardiol. (2020) 27:NP6–NP15. doi: 10.1177/2047487313501120, PMID: 23928568

[45] Estruch R Martínez-González MA Corella D Salas-Salvadó J Ruiz-Gutiérrez V Covas MI. Effects of a Mediterranean-style diet on cardiovascular risk factors: a randomized trial. Ann Intern Med. (2006) 145:1–11. doi: 10.7326/0003-4819-145-1-200607040-00004 16818923

[46] Mohammadifard N Salehi-Abargouei A Salas-Salvadó J Guasch-Ferré M Humphries K Sarrafzadegan N. The effect of tree nut, peanut, and soy nut consumption on blood pressure: a systematic review and meta-analysis of randomized controlled clinical trials. Am J Clin Nutr. (2015) 101:966–82. doi: 10.3945/ajcn.114.091595 25809855

[47] Tindall AM Johnston EA Kris-Etherton PM Petersen KS. The effect of nuts on markers of glycemic control: a systematic review and meta-analysis of randomized controlled trials. Am J Clin Nutr. (2019) 109:297–314. doi: 10.1093/ajcn/nqy236, PMID: 30722007 PMC7307437

[48] Vajdi M Karimi A Tousi AZ Hosseini B Nikniaz Z Farhangi MA. Association between plant-based diets and metabolic syndrome in obese adults from Iran: a cross-sectional study. BMC Endocr Disord. (2023) 23:109. doi: 10.1186/s12902-023-01358-7, PMID: 37193979 PMC10186771

[49] Rehm CD Peñalvo JL Afshin A Mozaffarian D. Dietary intake among US adults, 1999–2012. JAMA. (2016) 315:2542–53. doi: 10.1001/jama.2016.7491, PMID: 27327801 PMC6287627

[50] Batterham MJ Neale EP Nikodijevic CJ Probst YC Tapsell LC. Nut consumption in a representative survey of Australians: a secondary analysis of the 2011–2012 National Nutrition and physical activity survey. Public Health Nutr. (2020) 23:3368–78. doi: 10.1017/S1368980019004117, PMID: 32151295 PMC7737041

[51] U.S. Department of Agriculture. Agricultural Research Service USDA National Nutrient Database for standard reference, release 28. Beltsville, MD; (2016). Available at: http://www.ars.usda.gov/ba/bhnrc/ndl.

